# Communicating the Experience of Chronic Pain and Illness Through Blogging

**DOI:** 10.2196/jmir.2002

**Published:** 2012-10-23

**Authors:** Pamela Katz Ressler, Ylisabyth S Bradshaw, Lisa Gualtieri, Kenneth Kwan Ho Chui

**Affiliations:** ^1^Pain Research, Education and Policy ProgramDepartment of Public Health and Community MedicineTufts University School of MedicineBoston, MAUnited States; ^2^Health Communication ProgramDepartment of Public Health and Community MedicineTufts University School of MedicineBoston, MAUnited States; ^3^Department of Public Health and Community MedicineTufts University School of MedicineBoston, MAUnited States

**Keywords:** Blogging, narrative medicine, disease management, Internet, pain, chronic illness, survey, psychosocial support systems, holistic health, selfcare

## Abstract

**Background:**

Although more individuals are sharing their experiences with chronic pain or illness through blogging (writing an Internet web log), research on the psychosocial effects and motivating factors for initiating and maintaining a blog is lacking.

**Objective:**

The objective was to examine via online questionnaire the perceived psychosocial and health benefits of blogging among patients who use this media to communicate their experience of chronic pain or illness.

**Methods:**

A 34-item online questionnaire was created, tested, and promoted through online health/disease forums. The survey employed convenience sampling and was open from May 5 to July 2, 2011. Respondents provided information regarding demographics, health condition, initiation and upkeep of blogs, and dynamics of online communication. Qualitative data regarding respondents’ blogging experiences, expectations for blogging, and the perceived effects from blogging on the blogger’s health, interpersonal relationships, and quality of life were collected in the form of written narrative.

**Results:**

Out of 372 respondents who started the survey, 230 completed the entire questionnaire. Demographic data showed survey respondents to be predominantly female (81.8%) and highly educated (97.2% > high school education and 39.6% with graduate school or professional degrees). A wide spectrum of chronic pain and illness diagnoses and comorbidities were represented. Respondents reported that initiating and maintaining an illness blog resulted in increased connection with others, decreased isolation, and provided an opportunity to tell their illness story. Blogging promoted accountability (to self and others) and created opportunities for making meaning and gaining insights from the experience of illness, which nurtured a sense of purpose and furthered their understanding of their illness.

**Conclusions:**

Results suggest that blogging about chronic pain and illness may decrease a sense of isolation through the establishment of online connections with others and increases a sense of purpose to help others in similar situations. Further study involving a larger sample size, a wider range of education levels, and respondents with different types and magnitudes of illnesses will be needed to better elucidate the mechanism of the observed associations in this understudied area.

## Introduction

Chronic pain and illness are universal disorders. Globally, over 1.5 billion individuals suffer from chronic pain [1]. In the United States, 1 out of every 2 adults, or 133 million individuals, are living with at least one chronic disease [2], and 1 in 10 Americans live with severe chronic pain. Although we have made great strides in pain and disease management, chronic pain and illness remain costly health burdens and challenges for clinicians, patients, families, and society. In the United States alone, 25 million individuals report major limitations in daily living due to chronic disease [2]. Recent data suggest that 116 million US adults suffer from chronic pain creating an economic burden of $560 to $635 billion annually [1]. Advances in medical treatment allow many individuals to survive for a significant time with diseases that previously would have quickly resulted in death. Patients and families face challenges managing the physical as well as the social and emotional consequences of chronic pain and illness for extended lengths of time, often years or decades. To address this new paradigm of medical care for chronic conditions, it will become increasingly necessary for our health care system to identify and adopt effective interventions that could provide support to patients and families throughout their journey of diagnosis, treatment, and management.

Disciplines across the health care professions, including medicine, nursing, psychology, social work, and sociology, are actively engaged in understanding the psychosocial and emotional consequences of chronic pain and illness [3]. Methods of creating reflection, connection, and finding meaning and understanding through use of illness narratives are advocated for patients and family members. Extensive medical and social science research has examined the use of patient narratives as methods of telling the story of illness and health [4-8]. Review of the literature on expressive writing suggests there are psychological and physical benefits associated with patients engaging in the process of expressive writing [9-15]. In her book, *Narrative Medicine: Honoring the Stories of Illness*, Charon [16] describes the experience of patients and families as they enter the divide between the sick and the well, a separation that often feels huge and unbridgeable to those navigating a new way of being:

“These divides between the sick and the well are unspeakably wide. Leveraged open by shame, rage, loss, and fear, these chasms can be unbridgeable. And yet, to get better, the patient needs to feel included among those who are not ill. The sick person needs to continue to be, somehow, the self he or she was before illness struck. For the sick patient to accept the care of well strangers, those strangers have to form a link, a passage between the sick and the healthy who tender care.”

Chronic pain and illness may have an isolating effect on individuals, changing their perceived roles in society and challenging their ability to find meaning in life and their illness. Creating connections between the world of the sick and world of the well can be important in the positive psychosocial functioning of individuals.

Individuals with chronic pain or illness may have various purposes in articulating illness narratives. Perceived benefits may be to acknowledge and validate the experience of pain and illness, express beliefs and values, create a temporal nature of the experience of disease, and make visible the often-invisible nature of pain. Communicating the experience of pain and illness may facilitate sharing between teller and listener, allow for reflection on the experience, and acknowledge the emotional and suffering component of pain. Bridging this chasm, as Charon suggests, may be an important part of finding a sense of wellness within illness.

Traditionally, communicating the experience of pain or illness was oral or written on paper. As Pennebaker [11] and others have suggested, expressing emotional or traumatic experiences with the written word through the process of expressive writing, may have therapeutic benefit for individuals. Although expressive writing in medicine has been researched over the past decades, the digital communication platform of the Internet has expanded the ability to express emotional experiences of pain or illness to a larger, real-time audience and for those experiences to not only to be expressed, but to be shared and commented on by others. Use of Internet-based tools such as blogs (web-based journals with user-generated content) allow this expression to take place.

The Pew Internet study found that 78% of adults in the United States are online [17]. A digital divide of Internet usage exists between individuals with and individuals without chronic disease. Internet users who report no chronic disease are more likely (81%) than those with one or more chronic illnesses (61%) to be online [18]. However, once online, having a chronic illness increases the likelihood of interacting through social media, such as blogs, to share knowledge with peers. When other demographic factors are controlled, having a chronic illness significantly increases the probability of contributing to a blog or online discussion [18]. For those with chronic disease who consume health information online, 37% responded that they read other’s commentaries or medical/health experiences on online newsgroups, websites, or blogs, and 6% have posted health comments to a blog. As a form of online community, blogs function as platforms for patients with chronic pain and illness to share information, practical advice, and emotional support [18]. However, research on their attitudes and perceived benefits of blogging has been lacking. By using tools such as blogs, patients and families may find a new way to bridge this divide between the sick and the well. Illness blogs may allow a patient to articulate his or her narrative with the advantage of online interaction with others—family, friends, and other patients with similar health concerns. Illness blogs have similarities with traditional forms of illness narrative; however, features of blogs, in particular the ease of sharing or commenting, are dissimilar to illness narratives. Narrative writing has a long history of being self-initiated and directed, some of its recent attention has been its effectiveness as a directed activity, guided in part by health care professionals as a therapeutic intervention. No research to date has indicated whether illness narratives through blogging are potentially adaptable as such a therapeutic process.

The goal of this study was to explore the use of patient illness blogs as a means of communicating the experience of chronic illness and pain and to articulate their unique set of benefits and barriers. We surveyed current illness bloggers to understand their self-perceived psychosocial and health effects associated with the blogging activity. We hypothesized that communicating the experience of illness through blogging may provide perceived positive psychosocial benefits to some patients with chronic pain or illness.

## Methods

As a primary step in the formulation of this project, a research theory in accordance with the discipline of nursing (the training of the first author) was identified. Nursing research has focused on the healing process within the context of the individual, environment, and community [19]. Nurse theorist Margaret Newman’s *Health as an Expanding Consciousness *was chosen as a framework for this study based on its assumption that health is not simply the absence of disease [20]. Further recognizing that health is an awareness or consciousness of the evolving interaction between the individual and his or her environment, and is possible regardless of presence or absence of disease. The theory states, “every person in every situation, no matter how disordered and hopeless it may seem, is part of the universal process of expanding consciousness—a process of becoming more of oneself, of finding a greater meaning in life, and of reaching new dimensions of connectedness with other people and the world” [20]. Newman’s theory defines this paradigm shift as moving away from only treatment of symptoms and moving toward recognition of pattern and connection by assisting individuals in viewing disease and disruption as part of a self-organizing dynamic process of health [21]. Theoretical constructs of finding greater meaning in life, reaching new dimensions of connectedness, and expanding views of illness and health were incorporated in the planning and development of a survey tool.

We developed a 34-item online questionnaire based on our theoretical constructs to examine the initiation, motivation, continuation, reinforcement, support, and process of blogging. We also queried our respondents on the impact of chronic illness and pain on their life and activities of daily living in the context of illness blogging and, if this changed over time, how it was manifested in their blogging. We asked how the illness or chronic pain impacted their life and queried them regarding their efforts at assisting other individuals as well as developing and maintaining support networks. Questions explored psychosocial themes of coping, isolation, connection, and support. Furthermore, we asked for respondents’ interactions with health care providers about their blogging and their recommendations to others about blogging. Multiple choice, matrix, and open-ended essay or comment questions were incorporated on one scrollable screen with the goal of optimizing the depth of responses. All respondents answered the same questionnaire; no adaptive design or timing was employed. Question order was not randomized because some questions followed directly from others. Respondents were also allowed to skip to the next question without providing an answer. The open survey was formatted with a web-based survey design tool, SurveyMonkey [22]. SurveyMonkey uses secure sockets layer (SSL) data encryption and it recognizes and prevents repeated access based on Internet protocol (IP) addresses. A draft questionnaire was reviewed and pretested by a sample of three bloggers who provided qualitative assessments, allowing for small refinements to the survey, and the resulting questionnaire was used (Appendix 1).

After receiving approval from the Tufts Medical Center Institutional Review Board (IRB), the recruitment script and URL survey link was circulated among online health forums and online illness support groups, developing a compounding “snowball” convenience sample from Boston-centric academic and social media contacts. The authors also circulated the request for participation on the social media sites Twitter, Facebook, WEGO Health [23] (forums included chronic pain, fibromyalgia, and health bloggers subgroups of WEGO Health), and LinkedIn (American Pain Society discussion list). The disseminated request was in the following format (or abbreviated to fit into social media formats): “Do you blog about your illness or do you know someone who does? Help researchers at Tufts University School of Medicine better understand patient blogging by participating in a short online survey. Please forward the survey link https://www.surveymonkey.com/s/TuftsPatientBloggingSurvey to your friends and colleagues.” Although not promoted in the requests, a drawing for a prize of two $25 online shopping gift certificates was promoted on the consent page, which also included information about the purpose of the study, approval from the IRB, and the investigators’ contact information. The survey remained open for participation from May 5 to July 2, 2011, resulting in 230 completed surveys by the end of the survey timeframe.

Quantitative responses to the questionnaires were tallied and tabulated; text responses were analyzed qualitatively in an iterative process, using grounded theory principles to develop primary and subthemes [24]. The first two authors (PKR and YSB) separately reviewed the narrative responses and agreed on primary and secondary themes within each question. Categorizations were reviewed, differences were discussed, and then they were re-sorted independently until agreement was reached. Quotations were selected to represent the core themes, excluding direct quotes containing specific details or identifying references. We adhered to the Checklist for Reporting Results of Internet E-Surveys (CHERRIES) in reporting the results of this online questionnaire [25]. No weighting scheme was used.

## Results

### Response Statistics

Out of the 526 submissions, 379 agreed to consent, 1 disagreed, and 146 did not answer the consent question. Out of the 379 consented submissions, 149 did not answer any questions but applied for the prize with an email address, and hence were excluded from the analysis. The effective sample size is 230 and the completion rate is 60.7% (230/379). Due to the open sample frame, no response rate was computed.

### Characteristics and Blogging Behaviors

As part of the survey design, respondents were permitted to skip any question within the survey, so the number of responses varied by question. Table 1 shows the demographics of the participants.

Respondents identified the disease or conditions prompting the blog. The varied responses had common features of chronicity and potential for associated suffering and pain. Diseases represented included cancer (non-specified, ovarian, breast, and leukemia), fibromyalgia, non-specified chronic pain conditions, diabetes, Addison’s disease, bipolar disorder, celiac disease, trigeminal neuralgia, Parkinson’s disease, systemic lupus erythematosus (SLE), rheumatoid arthritis, Sjögren’s syndrome, schizophrenia, cystic fibrosis, cerebral palsy, as well as other chronic diseases and syndromes. Several respondents reported comorbidities, such as fibromyalgia and depression, bipolar disease and chronic pain, cerebral palsy and trigeminal neuralgia, breast cancer and chronic depression, and diabetes and clinical depression.

Table 2 shows blogging-specific behaviors of respondents. The survey asked questions regarding the initiation and maintenance of the participants’ blogs. Most bloggers (60.0%) had initiated their blogs on their own, and the majority of the blogs (87.8%) were public and searchable on the Internet. Although 64.1% of the bloggers used their own names in blogging, 35.8% chose to protect their identity by use of a pseudonym/pen name or by blogging anonymously. Although 89.7% of the respondents shared their blogs with friends and family members, less than half (42.1%) shared their blogs with their health care providers.

#### Theme analysis

Open-ended questions (with unlimited space in which to answer) explored respondents’ attitudes, motivations, and explanations. These narrative responses were qualitatively analyzed and grouped into primary themes according to the major ideas and concerns expressed.

The 57.9% of respondents who had not shared their blogs with their health care providers were asked why they made this choice. Their narrative answers provided information that was sorted into four primary themes: (1) negative concern (ie, respondent expressed concern that a negative outcome would result if the blog was shared), (2) perceived lack of provider interest and/or time to read the blog, (3) interest or intention to share the blog with their health care provider, and (4) desire to write freely about the illness experience.

In primary theme 1, negative concern*, *five subthemes were identified: (1) fear of provider judgment of patient feelings—concern of negative or pejorative judgment by the health care provider for the feelings expressed in the blog, (2) fear of provider judgment of patient behavior—concern that the provider would not approve of patient’s behaviors and/or lifestyle choices, (3) confidentiality/negative impact, (4) worry over self-editing, and (5) awkwardness in sharing negative opinions of health care provider and care (Table 3).

**Table 1:** Demographics of the survey participants (N = 230).

Demographic		n	%
**Gender**			
	Female	172	81.5
	Male	39	18.5
**Age group**			
	18-25	19	9.0
	26-39	71	33.8
	40-55	89	42.4
	56-65	25	11.9
	66-75	6	2.9
**Education**			
	Some high school	3	1.4
	Graduated high school or GED	3	1.4
	Some college	36	17.0
	Graduated college	86	40.6
	Graduate or professional degree	84	39.6

**Table 2:** Blogging-related behaviors of the survey participants (N = 230).

Blogging behavior		n	%
**Who suggested blogging? (multiple choices allowed)**			
	No one	138	60.0
	Family member	33	14.3
	Friend	50	21.7
	Work colleague	10	4.3
	Support group	8	3.5
	Health care provider	6	2.6
**When did you start blogging?**			
	Before 2007	40	17.5
	2007	17	7.5
	2008	26	11.4
	2009	48	21.1
	2010	65	28.5
	2011	32	14.0
**Blog is...**			
	Open to public	194	87.8
	Private, only individuals with password can access	27	12.2
**Blog is written under...**			
	The author’s own name	143	64.1
	A pen name or pseudonym	67	30.0
	An anonymous identity	13	5.8
**Blogging frequency**			
	More than once a day	9	4.3
	Once a day	19	9.0
	A few times a week	91	38.6
	Once a week	41	19.5
	Every few weeks	35	16.7
	Once a month or less	25	11.9
**Blogging frequency over time has...**			
	Increased	75	33.5
	Stayed the same	68	30.4
	Decreased	81	36.2
**Have viewers ever left written comments?**			
	Yes	206	91.6
	No	13	5.8
	Not sure	6	2.7
**Blogging platform/hosting used**			
	Blogger (by Google)	103	54.8
	WordPress	71	37.8
	CaringBridge	10	5.3
	Others	4	2.1
**Do you read other people’s health/illness blogs?**			
	Yes	214	95.1
	No	11	4.9
**Have you contributed comments on other’s blogs?**			
	Yes	202	89.0
	No	25	11.0
**Shared own blog with...**			
	Family members or friends	192	89.7
	Health care provider(s)	90	42.1
**Participated in any online health/illness or patient communities?**			
	Yes	151	72.9
	No	56	27.1
**Has reading blog and blogging changed one’s sense of connection with others?**			
	Yes	163	76.5
	No	22	10.3
	Not sure	28	13.1
**Other social media used (multiple choices allowed)**			
	Facebook	195	84.8
	Twitter	162	70.4
	YouTube	108	47.0
	Foursquare	26	11.3
	Others (LinkedIn, Tumblr, WEGO Health, etc)	43	18.7

A minority of the bloggers (42.1%) had shared their blogs with their health care providers. The health care providers’ responses to the illness blogs varied from positive to negative. Patient’s perceptions of health care provider’s reactions upon sharing illness blogs were grouped into three primary themes: (1) positive response, (2) negative response, and (3) neutral or indeterminate response (Table 4).

Motivation to start an illness blog clustered around three main themes: (1) reflection, (2) communication, and (3) connection with others. Additional subthemes surrounding the primary theme, connection with others, were identified as: (1) loneliness/isolation and (2) sharing knowledge/education. A number of respondents mentioned being able to “talk openly” and not being “so alone” with their disease as a positive impact of blogging about their illness. The words “share” and “sharing” were used often in the respondents’ answers (Table 5).

When questioned whether blogging had made a difference in dealing with the challenges of chronic pain or illness, respondents indicated positive changes. Responses were grouped and the following themes identified: (1) reframing/ability to gain broader perspective on illness, (2) identifying patterns of illness, (3) providing an expressive outlet, (4) support/feedback, ( 5) accountability, and ( 6) helping to cope with illness. Respondents mentioned an ability to frame the impact of their illness differently and notice other aspects of their lives. Blogging provided an outlet of expression for some respondents, as well as a way to identify patterns of illness. Accountability was also a theme that was repeatedly mentioned, both accountability to one’s self—with the implied commitment to blog—and accountability to others. Respondents reported that their overall ability to cope with chronic illness and pain was impacted positively by blogging (Table 6). Support and feedback from others who posted comments were also important factors for many respondents.

Blog-posting frequency and blog content evolved over time for many respondents. The initial use of a blog as a communication device for updating friends and family about a medical condition often evolved to helping educate others with the same disease or transitioning into a method of advocacy, mentorship, support, connection, and resolution with self and others. Identified themes regarding blog-posting frequency were (1) sense of evolving purpose (with subthemes of mentoring and advocacy), (2) making meaning of one's experience by understanding one’s illness more completely, and (3) resolution, indicating an endpoint or shift in one’s narrative (Table 7).

**Table 3:** Respondents’ reasons for not sharing their blogs with their health care providers.

Themes identified		Quotes	
**Theme 1.1: Negative concern**		
	A. Fear of provider judgment of patient feelings	“I don’t trust doctors to respect my feelings and opinions.”
		“I would feel awkward doing so.”
	B. Fear of provider judgment of patient behavior	“Would be reluctant as they would probably disagree with my lifestyle choices.”
	C. Privacy concern/negative impact	“Concern for personal privacy.”
		“I wonder if that would ever have a negative impact on my insurance rates or insurability.”
	D. Worry over self-editing	“I might edit myself if I knew they were reading.”
	E. Awkwardness in sharing negative opinions of health care provider and care	“They think they know everything and they don’t and some of the blog is about them.” “I did not share my blog with my doctor’s because I sometimes wrote about frustrations with the practice in general...and felt it might have been awkward to share.”
Theme 1.2: Lack of interest or time by health care provider to read patient blogs		“Doctors are not even close to interested in what I have to say, much less what I write!” “They’ve never seemed interested when I casually mentioned blogs, so I never mentioned that I write one myself.” “No, my doctor just provides medication and doesn’t discuss much with me.”
Theme 1.3: Intention to share		“I plan to the next visit with her. This was one idea we came up with to overcome my fears.” “I never specifically talked about my blog because it never came up in conversation. I would if it came up or she asked about it.” “It hasn’t really come up yet, but I suspect it will, the next time I visit.”
Theme 1.4: Desire to write freely about illness experience		“I want to be able to say whatever I want even about them.” “The content on my blog is personal and very opinionated.” “I wanted the freedom to post about my medical experiences without worrying about what my doctor would think.”

Sense of connection with others is an integral part of health and well-being for humans. Health as Expanding Consciousness theory posits that health is a dynamic quality in our evolving sense of interaction and connection with self and others, and a process of discovering meaning and understanding. Chronic illness and pain can separate an individual from their usual forms of interaction and meaning (ie, negative impact on quality of life measurements, such as work, family relationships, independence, and mobility) and new forms of interaction and meaning need to be cultivated in order to support positive psychosocial health. The primary themes of (1) helping others, (2) decreasing sense of isolation, (3) sharing of experiences, and (4) finding a sense of community and authentic voice within an individual’s co-existence with a chronic illness were articulated through many of the responses to the question: “Has writing or reading patient blogs changed your sense of connection with others?” (Table 8).

Although most of the respondents did recommend blogging for someone with a chronic illness, some did express that blogging necessitated a time commitment and may not be appropriate for everyone. Of those who did recommend blogging, their reasons for recommendation were grouped into themes of (1) gaining understanding/perspective of one’s chronic illness pattern, (2) emotional release, (3) decreased isolation, and (4) communication with others. A fifth theme of “would recommend with caveat” is also included (Table 9).

Privacy and disclosure, mental health issues, emotional vulnerability (discomfort/stress and anger were identified as subthemes), stage of disease (newly diagnosed), and stigma surrounding certain illnesses were the main themes of concern expressed when asked the question: “Are there circumstances when blogging should not be recommended?” The majority of those answering this question responded that there were circumstances when they would not recommend blogging to an individual (Table 10).

**Table 4:** Responses from health care providers upon knowing about the blog.

Themes identified	Quotes
Theme 2.1: Positive response	“Some read it, some don’t because of regulations. [They] found it very helpful for people in the same situation.”
	“Very well received.”
	“Positively, as I have great health care providers.”
Theme 2.2: Negative response	“Absolutely no interest.”
	“They don’t really like it. One clinic even forbid us to go there because of something I published in my blog.”
Theme 2.3: Neutral or indeterminate response	“I don’t know that he has ever visited, but I believe he thinks it is a good thing.”
	“Seemed to be a ‘passing’ interest in it.”

**Table 5:** Motivations for starting the blog.

Themes identified		Quotes	
Theme 3.1: Reflection		“Mostly to sort out my thoughts.”
		“I thought it’d help me deal with the pain, by releasing it emotionally.”
		“The suicide of a friend.”
Theme 3.2: Communication		“I wanted a forum where I could talk honestly about the symptoms I was experiencing.”
		“Need for support and to streamline communications.”
		“I wanted to be able to share what was happening to me with friends and family but knew that I wouldn’t have the time/energy to communicate with everyone individually.”
**Theme 3.3: Connection with others**		
	A. Loneliness, isolation	“Needing to find others who lived in chronic pain so I don’t feel so alone in my pain.”
		“I felt alone and isolated. And then when I started getting comments I realized I was helping other people cope.”
		“Share our reality with others.”
	B. Education, knowledge sharing	“Goal is to contribute in whatever way I can to knowledge about chronic pain as a real disease with real physical consequences.”
		“Share knowledge on health advocacy.”
		“I blog to not only talk about things I am going thru but hopefully to help others as well.”

**Table 6:** Did blogging change how one deals with the challenges of chronic illness or pain?

Themes identified	Quotes
Theme 4.1: Reframing/ability to gain broader perspective on illness	“Mostly the writing helps me see possibilities open to me. I am more active as a patient in the direction of my own care.” “It has helped me to process information and be more objective, which is often helpful.” “It helps me get perspective on my own situation in a way I didn’t expect.”
Theme 4.2: Identifying patterns of illness	“It has made me more aware of issues I had not considered. By keeping a log, I can pick up patterns I may have missed.” “I find it easier to identify triggers and patterns.”
	
Theme 4.3: Expressive outlet	“It has become an online journal where I have an outlet to discuss what I am feeling, emotionally and physically and it has helped my friends and family be kept up to date on things.”
	“Starting my blog has given me an outlet to express myself, and since I have started my blog I feel better about myself.”
	“Much needed infusion of positive energy.”
Theme 4.4: Support and feedback	“It has helped me enormously—processing and rationalizing, sharing and having feedback and support.” “It made me feel more connected to people, especially when I met people on Twitter and Tumblr that I could talk to about my chronic illness.” “I have met others with my condition or a similar one, learned about both ideas for coping with and the potential progression of my conditions, and developed great networks to connect people with common conditions.” “It’s helped me realize that I’m not alone.”
Theme 4.5: Accountability	“I felt it made me more accountable to follow up on things with my doctors because I knew people would ask about them.”
	“I am a much more informed patient and I’m also much more comfortable talking to others about diabetes. I no longer feel so isolated.”
	“Writing a blog has made me more aware of my habits and also more accountable. Additionally, I have been able to look at the positive aspects of dealing with illness largely because of my blogging and the opportunities it has created for me.”
Theme 4.6: Ability to cope with illness	“Helped me cope with the anxiety.” “It makes a positive impact on my ability to deal with my care.” “It’s actually made me realize that my illness is impacting my life less than I had imagined.”

**Table 7:** Evolution of the objectives of blogging.

Themes identified		Quotes
**Theme 5.1: Sense of evolving purpose**		
	A. Mentoring	“As my knowledge has increased I have become more of a mentor to others.”
		“Expanded the topics and added guest bloggers.”
		“The desire to make something good out of the struggles that I had faced and was facing at that time.”
	B. Advocacy	“It started out with just my personal stories and then I started sharing research and attempted to be an advocate.”
		“My blog content has changed mostly as I have a desire to spread more awareness and create a message that others will better relate to and understand.”
		“I take more of a patient advocacy stance.”
Theme 5.2: Making meaning of the experience of illness/understanding illness		“As my networking has grown my knowledge has also grown but so has my understanding of my own disability.”
		“I’ve come to terms with being ill, so there’s less ‘oh goodness it’s weird and scary encountering (insert fact of disabled life).’ I’ve become more political. And there’s more stuff without a disability angle.”
		“I believe I’ve opened up and been more honest over time.”
Theme 5.3: Resolution		“I have taken my blog down now. Once I was over the worst of my medical issues I no longer felt it appropriate to continue it.”
		“Not posting as much as I’m dealing with everything better.”

**Table 8:** Sense of connection cultivated through blogging.

Themes identified	Quotes
Theme 6.1: Helping others	“First I was helped, now I am helping...a reminder that I *am* part of the world.”
	“Cancer survivors are an amazing group, so eager to help each other.”
	“Reading about other breast cancer survivors is inspiring, sad, and makes me want to work harder to end this epidemic.”
Theme 6.2: Decreased sense of isolation	“By writing openly and in detail and having interaction with others it has enhanced my sense of interaction.”
	“It has helped me learn to talk to others about my issues more openly.”
	“I’m not alone! There are others out there who are in the same situation as me!”
Theme 6.3: Shared experience	“It helps me dealing with my own situation knowing that there are people in more or less the same situation.”
	“I feel there is a strong sense of understanding between people with a common disease as well as between people with different chronic disorders.”
	“It’s been wonderful connecting with others who really get what it’s like to live with chronic pain.”
Theme 6.4: Sense of community	“Enabled me to connect with others worldwide I would never have normally met. Enabled me to become closer to friends.”
	“There is a sense of ‘we’re all in this together’ with other bloggers who are faced with similar challenges, even if we face different illnesses; we often have a lot in common. It can be a great comfort.”
	“Realize that others are going through the same things as I am.”

**Table 9:** Reasons of recommending blogging.

Themes identified	Quotes
Theme 7.1: Gaining understanding/perspective	“Great way to work things out. Forces you to think about what the impact of the illness is (or is not!) on your life.”
	“It takes stress away and avoids an endless story repetition to those interested.”
Theme 7.2: Emotional release	“It’s a good release of emotions and you can set the level of privacy on each post so you can choose who sees what!”
	“It helps to write down your feelings and what you are going through.”
	“I think, if nothing else, it is a good outlet and a healthy way to share your feelings.”
Theme 7.3: Decreased isolation	“Certainly to people who enjoy writing, though, and to people who are socially isolated, although never blog *just* about illness.”
	“Yes, eases sense of isolation helps in staying on top of treatment changes, enables conversations that the healthy do not want to have.”
Theme 7.4: Communication with self or others	“It can help to connect with others in a similar situation and also help you understand yourself and your illness more.”
	“Healthy communication of pain is never wrong.”
	“I think it can have an impact on helping others to ‘get it.’”
Theme 7.5: Would recommend blogging with caveat	“If they are comfortable doing it. Privacy concerns are the 800-lb gorilla in the room.”
	“Only if they want to, because it can be really demanding. But I would recommend it because it will help them to become more empowered, informed and confident.”
	“Maybe a private blog? Or a blog within an online fibromyalgia community to feel safer.”

**Table 10:** Perceived situations in which blogging would not be recommended.

Themes identified		Quotes
Theme 8.1: Privacy/disclosure		“I would not recommend blogging for those who do not feel comfortable sharing personal information.”
		“Those with privacy concerns, such as health insurance tied to employment, need to be cautious. Those who would be harmed by public disclosure or being found by search engines.”
		“Anything published on the Internet stays there forever.”
Theme 8.2: Stage of disease (newly diagnosed)		“Maybe when still new to the diagnosis and searching for answers.”
Theme 8.3: Mental Illness		“If you are very unwell (mentally) and might say something that could ‘trigger’ someone else to do something harmful toward themselves or others, you should either not write until you are a bit better or censor what you write.”
		“Anyone with a mental illness who cannot handle negative comments should not blog.”
Theme 8.4: Stigma of illness		“When people are uncomfortable talking about their conditions. There are also regional issues, stigmas to think about. If you don’t like being found with a Google search of the condition and your name, don’t blog.”
**Theme 8.5: Emotional vulnerability**		
	A. Discomfort/stress	“If it would make the person feel uncomfortable sharing medical information or if it would stress them out too much.”
		“If it hurts someone else.”
		“When patient is too weak or under strong emotional distress.”
	B. Anger	“Don’t blog when angry. You’ll say things you’ll regret.”
		“Express anger aimed at another individual.”

## Discussion

This project contributes to formative research on illness blogging and provides information leading to a more thorough understanding of why individuals initiate and maintain an illness blog. This convenience sample of primarily well-educated women mirrors the population who are most engaged with social media; however, this is not fully representative of the overall online population with chronic disease, based on the Pew data [18]. The Pew Internet and American Life Project has found in their surveys of Internet and social networking usage in the United States that an adult with chronic illness is less likely to have Internet access compared to an adult without chronic illness (62% versus 81%) [18]. Approximately the same proportion of patients with and without chronic conditions either consume (57%) or generate (20%) online health information.

Overall, respondents provided information that illustrates how blogging assisted them in sharing, responding, reflecting, and evolving in their understanding and experience of their own health conditions and illness challenges. This evidence demonstrates how blogging may assist some patients in articulating and sharing their illness narratives. Although health care providers only suggested blogging to 6% of respondents, the positive elements identified by bloggers suggests therapeutic potential in this social media tool. Further research is needed to develop the exploratory data reported here and to explore patient characteristics, diagnoses, or conditions that might find such a process useful. Similar to evidence that journaling provides moderation of pain and increases self-management of chronic pain, it is possible that with further research, providers may be able to better identify safe and effective conditions for recommendation of illness blogging as well as understand methods to minimize negative effects of illness blogging. Continued research may find that development of blogging as a health care tool could be a beneficial intervention for some patients with chronic pain or illness.

Respondents provided longer, more reflective responses to the open-ended survey questions than expected, identifying perceived motivators and psychosocial benefits of blogging about one’s illness. Most bloggers started their blog on their own initiative, and this may suggest that this cohort already had strong illness coping strategies in place. A future research question to assess is whether introducing illness blogging by a health care provider would have a similar positive effect. Illness bloggers readily shared their blogs with friends and family, and the majority of the blogs were not password or otherwise protected from public view or search capabilities. In contrast to this open and public nature of illness blogging, a majority of respondents did not share their blogs with their health care providers. The perception that health care providers would be uninterested or lacked time, and had been hostile or were anticipated to be hostile toward the patient and/or the contents of the illness blog was expressed in a number of survey responses. Some respondents felt if their health care provider read or commented on their blog, it would stifle the free flow of emotions and ideas that blogging provides. This may be an issue to investigate further with health care providers as well as illness bloggers. The theme of accountability expressed by the bloggers was unanticipated, yet it is consistent with other responses of developing greater connections with others and deeper insight into one’s self through the reflective process of blogging. The accountability expressed appeared to be a motivating factor resulting in a sense of responsibility, purpose, and attempting to validate and gain understanding of the experience of illness by the process of initiating and maintaining a blog.

The creation of online connections with others by commenting and reading others’ illness blogs supports the concepts framed by the theory of Health as Expanding Consciousness, which posits that individuals strive to regain a sense of health by connecting with self and others, and finding growth, meaning, and purpose in life experiences. The process of blogging allows for the creation of real-time sharing of experience with family and a community of others in similar situations; in contrast, traditional journal writing does not permit this same process. Several survey respondents did remark on the sense of being “less alone” and less isolated by their illness when engaging in the process of illness blogging.

Limitations of the study included a convenience sample resulting in a narrow demographic cohort of self-selected, highly educated, English speakers (the survey was not translated into languages other than English), who were predominantly women. This may have been due to active participation in the survey by online communities focused on breast cancer and fibromyalgia. A sample more closely representing the population with chronic illness and pain will need to be developed to further explore blogging issues, while accounting for evidence that education and income levels of bloggers tend to be higher than the general population [26]. Although women with chronic disease are proportionally more active in generating Internet content, such as blogs, further studies may want to include bloggers with more gender-neutral conditions, such as lung or colon cancer, heart disease, and diabetes. The matrix model of our survey design may have been a specific limitation because the question regarding the impact of their illness limited respondents’ ability to express if an item had both negative and positive impacts on their disease or illness which may have limited the ability of respondents to fully answer some questions. Financial aspects of illness were not addressed in this project; these might impact patients’ experiences and should be included in future studies.

Although there are limitations in this formative research, we believe this study adds to the growing body of knowledge on digital communication (blogging) and how it may play a role in addressing the needs of individuals living with chronic pain or illness. The project hypothesis, that communicating the experience of illness through blogging may provide positive psychosocial benefits to some patients with chronic pain or illness, appears to be supported. Larger studies that incorporate a comparison group in a longitudinal setting need to be undertaken to generalize the benefits to a wider population.

Given the data from respondents, further research in this area is indicated in several directions. There is limited literature examining the intersection between illness narratives and blogs. Based on this data, blogging appears to decrease a sense of isolation through the establishment of online connections with others and increases a sense of purpose by assisting others facing similar situations. Although blogging consumes time and energy for the blogger, illness blogging seems to facilitate a shared reflective experience through the process of reading, writing, and commenting on illness blogs. Both Charon [4] and Pennebaker [8] describe the benefits of telling and retelling of a narrative to come to greater understanding and meaning of the event. The experience of blogging enables the blogger to narrate their own health story in real time with evolving understanding and meaning. Qualitative analysis will assist in understanding how blogging further relates to expressive writing and narrative. Health as Expanding Consciousness theory suggests that humans are seeking a sense of meaning and purpose out of disorder and hopelessness [20]. These benefits may be harder to obtain in traditional journaling or illness narratives because of the lack of real-time communication and the broad reach offered by the use of the Internet.

It is noteworthy that illness bloggers perceive a lack of interest by the health care community in their blogs. This may be an area for further research within the health care community. As we have moved forward with medical advances supporting the ability to live longer, the numbers of people living with chronic disease and pain have increased. Improving the quality of life for people with chronic disease and pain requires greater recognition of not only the physical manifestations of pain and disease, but also their psychosocial manifestations. This new paradigm of health care includes living with illness for extended lengths of time and a need for the individual and family to create new ways of understanding and processing the dynamic health-illness continuum. Charon [16] states that it is essential to find ways to bridge the divide between illness and health, to reflect and seek understanding of what has happened, and often this can be facilitated by storytelling and narratives. The Health as Expanding Consciousness theory suggests a need for finding patterns and meaning, and a connection with one’s self and others are necessary tasks to complete in moving toward health. The words of a survey respondent, “First I was helped, now I am helping...a reminder that I am part of the world,” describe the experience process that bloggers in this project reported. Perhaps the process of communicating the experience of chronic illness and pain through blogging may be one method to assist in moving toward a more complete, holistic model of health and healing by allowing individuals with chronic illness and pain to regain a place in the world.

## Multimedia Appendix 1

Communicating the Experience of Chronic Pain and Illness through Blogging Survey.

## Abbreviations

CHERRIES: Checklist for Reporting Results of Internet E-Surveys
IP: Internet protocol
IRB: Institutional Review Board
SSL: secure sockets layer
