# Supplementary material for: Communicating the Experience of Chronic Pain and Illness Through Blogging
Source: J Med Internet Res. 2012 Oct 23;14(5):e143. doi: 10.2196/jmir.2002 (PMC3510726; doi:10.2196/jmir.2002)
Supplement: Supplementary file 1 [file jmir_v14i5e143_app1.pdf]

Tufts University Medical School: Patient Blogging Survey

INFORMED CONSENT FOR PARTICIPATION

Thank you for taking part in a Tufts University School of Medicine graduate research project to examine patient blogging.

Purpose of the Study: The purpose of this study is to examine the use of patient blogs.

What will be done: You will complete a survey, which will take 10-15 minutes to complete. The survey includes questions about your blogging history. We also will ask for some demographic information (e.g., age, marital status,education level) so that we can accurately describe the general traits of survey participants.

Confidentiality: Your responses will be kept completely confidential. We will NOT know your IP address when you respond to the survey. We will ask you to include an e-mail address when you complete the survey so that we can enter you in the drawing for the gift certificate. However, your e-mail address will not be stored with data from your survey. You will be assigned a participant number, and only that number will appear with your survey responses.

Benefits of this Survey: You will be contributing to knowledge about patient blogs. In addition, you will be entered in a drawing for one of two $25.00 Amazon.com gift certificates. After we have finished data collection, we will conduct the drawing. Winners will receive the gift certificate via e-mail.

Risks or discomforts: No risks or discomforts are anticipated from taking part in this study. If you feel uncomfortable with a question, you can skip that question or withdraw from the study altogether. If you decide to quit at any time before you have finished the survey, your answers will NOT be recorded.

Decision to quit at any time: Your participation is voluntary; you are free to withdraw your participation from this study at any time. If you do not want to continue, you can simply leave the survey site. If you do not click on the "done" button at the end of the survey, your answers and participation will not be recorded. You also may choose to skip any questions that you do not wish to answer. If you click on the “done” button at the end of the survey and enter your email address, you will be entered in the drawing.

How the findings will be used: The results of the study will be used for scholarly purposes only. The results from the study will be presented in educational settings and at professional conferences, and the results might be published in a professional journal.

Contact information: If you have concerns or questions about this study, you may contact the Principal Investigator, Dr. Libby Bradshaw, (617) 636-6946, Co-Investigator, Pamela Ressler, RN, MS-PREP candidate (pamela.ressler@tufts.edu), or faculty preceptor, Dr. Lisa Gualtieri (l.gualtieri@tufts.edu). If you have questions about your rights as a research study subject, call the Tufts Medical Center and Tufts University Health Sciences Institutional Review Board (IRB) at (617) 636-7512. The IRB is a group of doctors, nurses, and non-medical people who review human research studies for safety and protection of people who take part in the studies. Federal law requires the IRB to review and approve any research study involving humans.

By beginning the survey, you acknowledge that you are at least 18 years old, have read this information, and agree to participate in this research, with the knowledge that you are free to withdraw your participation at any time without penalty.

I have read and understand the information presented about this survey and agree to voluntarily participate.

- ○ Yes
- ○ No

Tufts University Medical School: Patient Blogging Survey

When did you start your blog?

- 2011
- 2010
- 2009
- 2008
- 2007
- before 2007

What was the medical diagnosis(es) or symptoms that prompted you to begin to blog?

Do you blog under:

- own name
- pen name or pseudonym
- anonymously

Is your blog:

- Public (anyone can view it on the internet)
- Private (only shared with individuals who have access to it through a password)

Other (please specify)

How often do you post on your blog?

- more than once a day
- once a day
- a few times a week
- once a week
- every few weeks
- once a month or less

Other (please specify)

Tufts University Medical School: Patient Blogging Survey

How has the frequency of your blogging changed over time?

- ○ Increased
- ○ Decreased
- ○ Stayed the same

Do people write comments on your blog?

- ○ Yes
- ○ No
- ○ Not sure

What motivated you to start a blog?

Who suggested that you start a blog?

- ☐ No one
- ☐ Family member
- ☐ Friend
- ☐ Work Colleague
- ☐ Support group
- ☐ Healthcare provider

Other

Tufts University Medical School: Patient Blogging Survey

In what ways has your illness or diagnosis impacted your life?

	Positive Impact	Negative Impact	No Impact	Not Sure	N/A
Work	○	○	○	○	○
Family Relationships	○	○	○	○	○
Ability to move around	○	○	○	○	○
Social Interactions	○	○	○	○	○
Connection with others	○	○	○	○	○
Spirituality	○	○	○	○	○
Diet	○	○	○	○	○
Sleep	○	○	○	○	○
Pain	○	○	○	○	○
Level of Independence	○	○	○	○	○

Other (please specify)

Has your blog content changed over time? If so, in what way?

Do you read other people's health/illness blogs?

○ Yes

○ No

Do you contribute comments on other blogs?

○ Yes

○ No

Has writing a blog made a difference in how you have dealt with your illness? If so, how?

Have you shared your blog with family members or friends?

○ Yes

○ No

What benefits and/or barriers have you found in writing a blog?

Tufts University Medical School: Patient Blogging Survey

Have you shared your blog with your health care provider(s)?

- Yes
- No

If you have NOT shared your blog with your health care provider(s), is there a reason?

If you HAVE shared your blog with your health care provider(s), how was it received?

Has writing or reading patient blogs changed your sense of connection with others?

- Yes
- No
- Not sure

If yes, how?

Would you recommend blogging to others who are living with a chronic illness? Why or why not?

Are there circumstances when blogging should not be recommended? If yes, when?

What suggestions do you have for others who may want to start to blog about their illness?

Tufts University Medical School: Patient Blogging Survey

What blogging platform/hosting do you use?

- Wordpress
- Blogger(Google)
- CaringBridge
- MovableType
- CarePages

Other (please specify)

Besides blogging, what forms of social media do you use?

	Yes	No
Facebook		
Twitter		
YouTube		
FourSquare		

Other types of social media (please specify)

Do you promote your new blog posts on social media sites? If so, which ones?

Do you participate in any online health/illness or patient communities? If yes, which ones?

- Yes
- No

Specific health/illness or patient related online communities in which you participate:

Tell us a bit about yourself:

	18-25	26-39	40-55	55-65	66-75	75+
Age						

Gender

- Male
- Female

Tufts University Medical School: Patient Blogging Survey

Marital Status

- Single
- Living with a partner
- Widowed
- Divorced
- Married
- Prefer not to answer

Educational Level

- Some high school
- Graduated high school or GED
- Some college
- Graduated college
- Graduate or Professional degree
- Prefer not to answer

How do you identify your race/ethnicity?

Is there anything we haven't asked that you would like to share about your blog or yourself?

We appreciate your willingness to add to scientific research and knowledge on patient blogging.

If you would like to be entered in the drawing to win a $25 Amazon gift certificate, please enter your email address. Otherwise, please skip the next question and then press "done"

Thank you.

To be entered in the gift certificate drawing, please enter a contact email address. Email addresses will NOT be associated with survey results.

Email Address:

If you would like to share your blog URL with us, please enter it in the text box below. As with your email address, it will NOT be associated with survey results.
